# Dual Regulation of Cell Death and Cell Survival upon Induction of Cellular Stress by Isopimara-7,15-Dien-19-Oic Acid in Cervical Cancer, HeLa Cells *In vitro*

**DOI:** 10.3389/fphar.2016.00089

**Published:** 2016-03-31

**Authors:** Nadiah Abu, Swee K. Yeap, Ahmad Z. Mat Pauzi, M. Nadeem Akhtar, Nur R. Zamberi, Jamil Ismail, Seema Zareen, Noorjahan B. Alitheen

**Affiliations:** ^1^Department of Cell and Molecular Biology, Faculty of Biotechnology and Biomolecular Sciences, Universiti Putra MalaysiaSerdang, Malaysia; ^2^Laboratory of Immunotherapeutics and Vaccine (LIVES), Institute of Bioscience, Universiti Putra MalaysiaSerdang, Malaysia; ^3^Faculty of Industrial Sciences and Technology, Universiti Malaysia PahangKuantan, Malaysia

**Keywords:** HeLa, isopimara-7, 15-dien-19-oic acid, *Fritillaria imperialis*, antioxidant

## Abstract

The *Fritillaria imperialis* is an ornamental flower that can be found in various parts of the world including Iraq, Afghanistan, Pakistan, and the Himalayas. The use of this plant as traditional remedy is widely known. This study aims to unveil the anti-cancer potentials of Isopimara-7,15-Dien-19-Oic Acid, extracted from the bulbs of *F. imperialis* in cervical cancer cell line, HeLa cells. Flow cytometry analysis of cell death, gene expression analysis via cDNA microarray and protein array were performed. Based on the results, Isopimara-7,15-Dien-19-Oic acid simultaneously induced cell death and promoted cell survival. The execution of apoptosis was apparent based on the flow cytometry results and regulation of both pro and anti-apoptotic genes. Additionally, the regulation of anti-oxidant genes were up-regulated especially thioredoxin, glutathione and superoxide dismutase- related genes. Moreover, the treatment also induced the activation of pro-survival heat shock proteins. Collectively, Isopimara-7,15-Dien-19-Oic Acid managed to induce cellular stress in HeLa cells and activate several anti- and pro survival pathways.

## Introduction

Cancer is a complex disease that is not fully understood yet. Cervical cancer is among the most diagnosed type of cancer in women today. Statistically, around 1 out of 154 will be diagnosed for cervical cancer (Siegel et al., 2014). The mechanism of cell death and cell survival often intertwines and involves a lot of variables. There is a delicate balance that plays a major role in cell sustenance and the tilt can lean either way, especially in reacting to external substances (Fulda et al., 2010). Unfortunately, a viable treatment for treating cervical cancer is yet to be found. Natural-derived molecules have become a promising target in finding the cure for major diseases including diabetes, cancer, and Alzheimer.

*Fritillaria imperialis* or commonly known as “crown imperial” is a species of flower from the Liliaceae family (Khare, 2007). This species can be found in various parts of the world specifically Iran, Turkey, Afghanistan, and some parts of the Himalaya (Khare, 2007; Badfar-Chaleshtori et al., 2012). This plant is considered an ornamental plant due to its large and attractive flowers. It is also known to have several medicinal properties such as becoming a diuretic, treating hypotensive, cardiotonic, and spasmolytic (Khare, 2007). There are several interesting molecules that can be extracted from this plant especially steroidal alkaloids (Atta-ur-Rahman et al., 2002; Akhtar et al., 2003; Khare, 2007). Additionally, another class of molecules that can also be extracted from the *F. imperialis* is sesquiterpenes (Atta-ur-Rahman et al., 2005). Sesquiterpenes are a class of natural products possessing various biological activities such as antimycobacterial (Abourashed et al., 2011), antifungal (Al-Ja'fari et al., 2013), anti-inflammatory, apoptosis-inducing, and immunosuppressant activities (Qi et al., 2015). Most of the sesquiterpene lactones impart a wide-range of pharmacological effects, including anti-cancer and immunomodulatory action (Lu et al., 2009; Choi et al., 2011; Ivanescu et al., 2015), antimicrobial, antioxidant, anti-inflammatory, and antinociceptive activities (Sulaiman et al., 2010; Dahham et al., 2015).

Diterpene Isopimara-7,15-dien-19-oic acid can be isolated from the bulbs of *F. imperialis* plant. The only known activity this molecule has is the prolyl endopeptidase inhibition (Atta-ur-Rahman et al., 2005). Other biological or chemical properties of this molecule are yet to be discovered. Thus, the aim of this study is to understand the molecular mechanism of HeLa cells, the most used cervical cancer cell line, upon induction with isopimara-7,15-dien-19-oic acid *in vitro*.

## Materials and methods

### Plant material and purification of compounds

**Figure 1:**
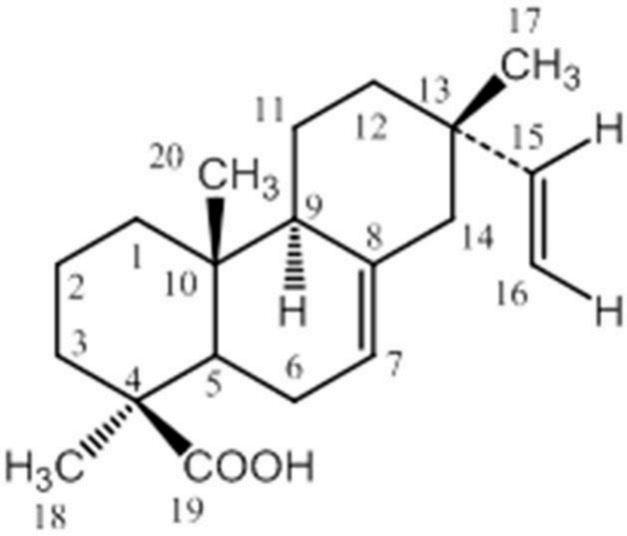
**The chemical structure of DIA isolated from the bulbs of ***Fritillaria imperialis*****.

Isopimara-7,15-dien-19-oic acid (Figure 1) was purified from a hexane fraction of Turkish plant *F. imperialis*. The hexane fraction was obtained as thick oil and subjected to column chromatography over silica gel by using acetone/petroleum ether as the solvent system. Isopimara-7,15-dien-19-oic acid was obtained as colorless prismatic crystals with melting point 159–160°C. The detailed extraction procedure and identification of isopimara-7,15-dien-19-oic acid were already published in our previous publication (Atta-ur-Rahman et al., 2005).

### Cell culture

HeLa cells were obtained from the ATCC Collection (ATCC, USA) and were maintained in RPMI-1640 (Sigma, USA) supplemented with 10% fetal bovine serum and 1% penicillin-streptomycin (Gibco, USA). The cells were incubated at 37°C equipped with 5% CO_2_.

### MTT

The MTT analysis was performed as a preliminary cytotoxic study for DIA against HeLa cells. Cells were seeded in a 96 well plate at a density of 0.8 × 10^5^ cells/ml overnight. Afterwards, the cells were treated with seven different doses of DIA ranging from 30 to 0.64 μg/mL and were left to incubate for 72 h. Upon reaching the allocated incubation time, 20 μl of 5 mg/mL of MTT (Sigma, USA) was added to each of the wells for 4 h at 37°C. Next, the media as well as the MTT were removed and 100 μl of DMSO was added to solubilize the resulting formazon crystals. Subsequently, the reading of the plate was obtained using a microplate reader (Bio-Tek Instruments, USA) at 570 nm.

### Cell cycle flow cytometry analysis

HeLa cells were seeded in 6 well plates at a density of 2.4 × 10^5^ cells/well overnight. The next day, 15 μg/mL of DIA was added into the designated wells and was left to incubate for 72 h in a humidified 37°C CO_2_ incubator. Afterwards, the cells were harvested, fixed in 500 μl of ice cold 80% ethanol and were stored in −20°C for 1 week. On the day of the analysis, the pellet was washed with 1 ml of PBS twice and was permeabilized and stained using 500 μl of RNAse-Propidium Iodide-PBS-Triton X100 for 15 min at room temperature. Next, the cells were analyzed using a FACS Calibur Flow Cytometry machine immediately (BD, USA).

### Annexin V-FITC flow cytometry analysis

This assay was performed according to the protocol set by the Annexin V/FITC kit by BD, USA. Similar to the cell cycle analysis, HeLa cells were seeded in 6 well plates at a density of 2.4 × 10^5^ cells/well overnight. The next day, 15 μg/mL of DIA was added into the designated wells and was left to incubate for 72 h in a humidified 37°C CO_2_ incubator. After the incubation time, the cells were harvested and washed twice with PBS. The cells were later stained with Annexin V-FITC and Propidium Iodide in 100 μl of 1X Binding Buffer. Then, the cells were analyzed using a FACS Calibur Flow Cytometry machine immediately (BD, USA).

### JC-1 flow cytometry analysis

The JC-1 analysis was done using the BD Mitoscreen Kit (BD, USA). Similar to the cell cycle analysis, HeLa cells were seeded in 6 well plates at a density of 2.4 × 10^5^ cells/well overnight. The next day, 15 μg/mL of DIA was added into the designated wells and was left to incubate for 72 h in a humidified 37°C CO_2_ incubator. After the incubation time, the cells were harvested and washed twice with PBS. Afterwards, the cells were stained and incubated with the JC-1 dye for 15 min at 37°C. Then the cells were washed twice with the provided washing buffer. The cells were later analyzed using a FACS Calibur Flow Cytometry machine immediately (BD, USA).

### cDNA microarray

Three sets of biological replicates of untreated HeLa and DIA-treated HeLa were prepared. HeLa cells were seeded in 6 well plates at a density of 2.4 × 10^5^ cells/well overnight. After 24 h, 15 μg/mL of DIA was added into the designated wells and was left to incubate for 48 h in a humidified 37°C CO_2_ incubator. After the incubation time, the cells were harvested and RNA was extracted using the QiagenRneasy Mini Kit (Qiagen, Germany). The quality of the RNA extracted was measured using the 2100 Bioanalyzer using a RNA Pico chip (Agilent, USA). In order to proceed to microarray, the RIN (RNA Integrity Number) should be more than eight. After all of the samples have passed the minimum requirement for microarray analysis the samples were then used for microarray. All of the samples were subjected to the SurePrint G3 Human Gene Expression 8x60K v2 microarray kit (Agilent Technologies, USA) according to manufacturer protocol, and scanned with Agilent DNA microarray scanner. The results from the microarray study has already been uploaded on the Gene Expression Omnibus with the accession number GSE72974.

### Differential expression analysis for microarray

The results from the microarray analysis were analyzed using the Genespring GX Software Version 13.1 (Agilent, USA). The differential expression comparison was made between the untreated HeLa cells and the DIA-treated HeLa cells. Final results were analyzed based on gene ontology with expression level having *p* < 0.05.

### Quantitative real-time PCR

To validate the results obtained from the microarray study, real-time PCR was performed on the same samples using different sets of primers. Around 1 μg of RNA from each of the samples were converted to cDNA using the Quantitect Reverse Transcription Kit according to the manufacturer's protocol (Qiagen, Germany). Then, real-time PCR was conducted using the SYBR Select Master Mix (Invitrogen, USA) on the iCycler IQ5 (Bio-rad, USA). Table 1 illustrates the name of the gene, accession number, and sequence of the primers used in this assay (http://pga.mgh.harvard.edu/primerbank/).

**Table 1:** **Accession number and the sequence of the primers used to validate the microarray results**.

**Gene name**	**Accession number**	**Sequence**
HMOX1	NM_002133.2	F: 5-AAGACTGCGTTCCTGCTCAAC-3
		R: 5-AAAGCCCTACAGCAACTGTCG-3
DDIT3	NM_001195057.1	F: 5-GAACGGCTCAAGCAGGAAATC-3
		R: 5-TTCACCATTCGGTCAATCAGAG-3
GPX3	NM_002084.3	F: 5-AGAGCCGGGGACAAGAGAA-3
		R: 5-ATTTGCCAGCATACTGCTTGA-3
GADD45A	NM_001924.3	F: 5-GAGAGCAGAAGACCGAAAGGA-3
		R: 5-CAGTGATCGTGCGCTGACT-3
ACTB	NM_001101.3	F: 5-AGAGCTACGAGCTGCCTGAC-3
		R: 5-AGCACTGTGTTGGCGTACAG-3
GAPDH	NM_002046.4	F: 5- GGATTTGGTCGTATTGGGC-3
		R: 5- TGGAAGATGGTGATGGGATT-3
18S RRNA	**HQ387008.1**	F: 5- GTAACCCGTTGAACCCCATT-3
		R: 5- CCATCCAATCGGTAGTAGCG -3

### Proteome profiler array ^TM^

The proteome profiler antibody array was employed to determine the effects of DIA on the activation of several cell stress-related proteins. This assay was done according to the manufacturer's protocol. The cell lysates were incubated with the designated membranes overnight at 4°C. The following day, the membranes were washed three times and were then incubated with the freshly prepared antibody cocktail for 2 h. Afterwards, the membranes were washed for three times again, before being incubated with the streptavidin-horseradish-peroxidase for 30 min at room temperature. Then, the membranes were developed under chemiluminescence conditions using the ChemiDOC XRS (Bio-rad, USA).

### Superoxide dismutase (SOD) and glutathione (GSH) quantification

Total proteins were extracted from the untreated HeLa and DIA-treated HeLa and were measured using the Bradford assay (Sigma, USA). For SOD, 100 μL of extracted protein was mixed with 200 μL of working solution (0.1 mol/L phosphate buffer, 0.15 mg/mL sodium cyanide in 0.1 mol/L ethylenediaminetetraacetic acid, 1.5 mmol/L nitrobluetetrazolium and 0.12 mmol/L riboflavin). On the other hand, GSH was quantified using Glutathione assay kit (Sigma, USA), where 10 μL of protein was added with 150 μL of working solution (1.5 mg/mL DTNB, 6 U/mL glutathione reductase, and 1 × assay buffer). After 5 min of incubation, 50 μL of NADPH solution (0.16 mg/mL) was added to the mixture. The absorbance for SOD and GSH were measured using ELISA plate reader (Bio-Tek Insturments, USA) at respective wavelengths of 560 and 420 nm.

### Statistical analysis

All experiments were done in three biological replicates and expressed as mean ± standard deviation. Results with statistical significant (*p* < 0.05) was assayed by student *t*-test comparing to the untreated control.

## Results

### DIA inhibited the proliferation of HeLa cells and induced apoptosis *in vitro*

**Figure 2:**
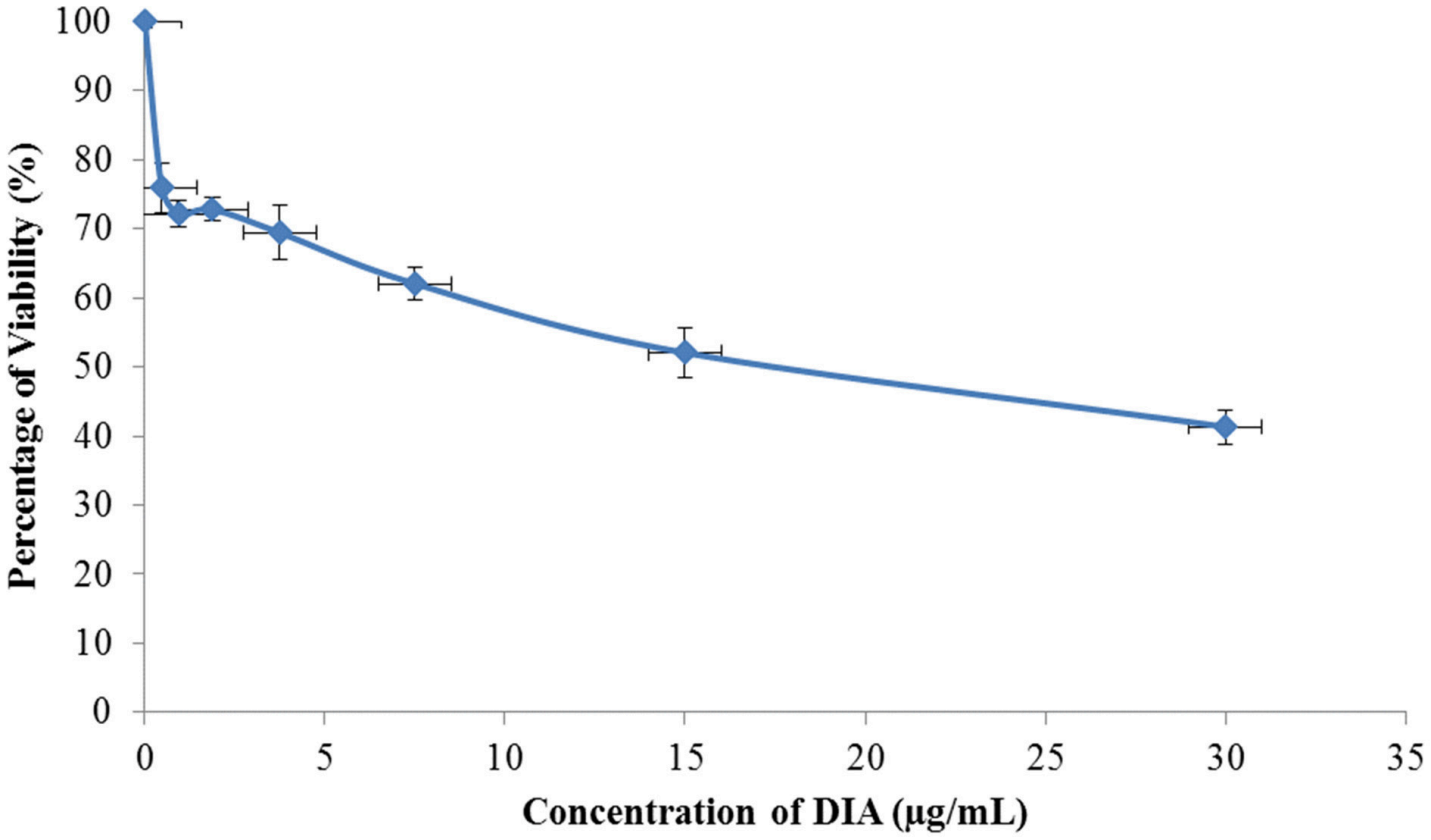
**MTT analysis of HeLa cells after being treated with DIA for 48 h at 30 μg/mL followed by seven 2-serial dilutions**.

**Figure 3:**
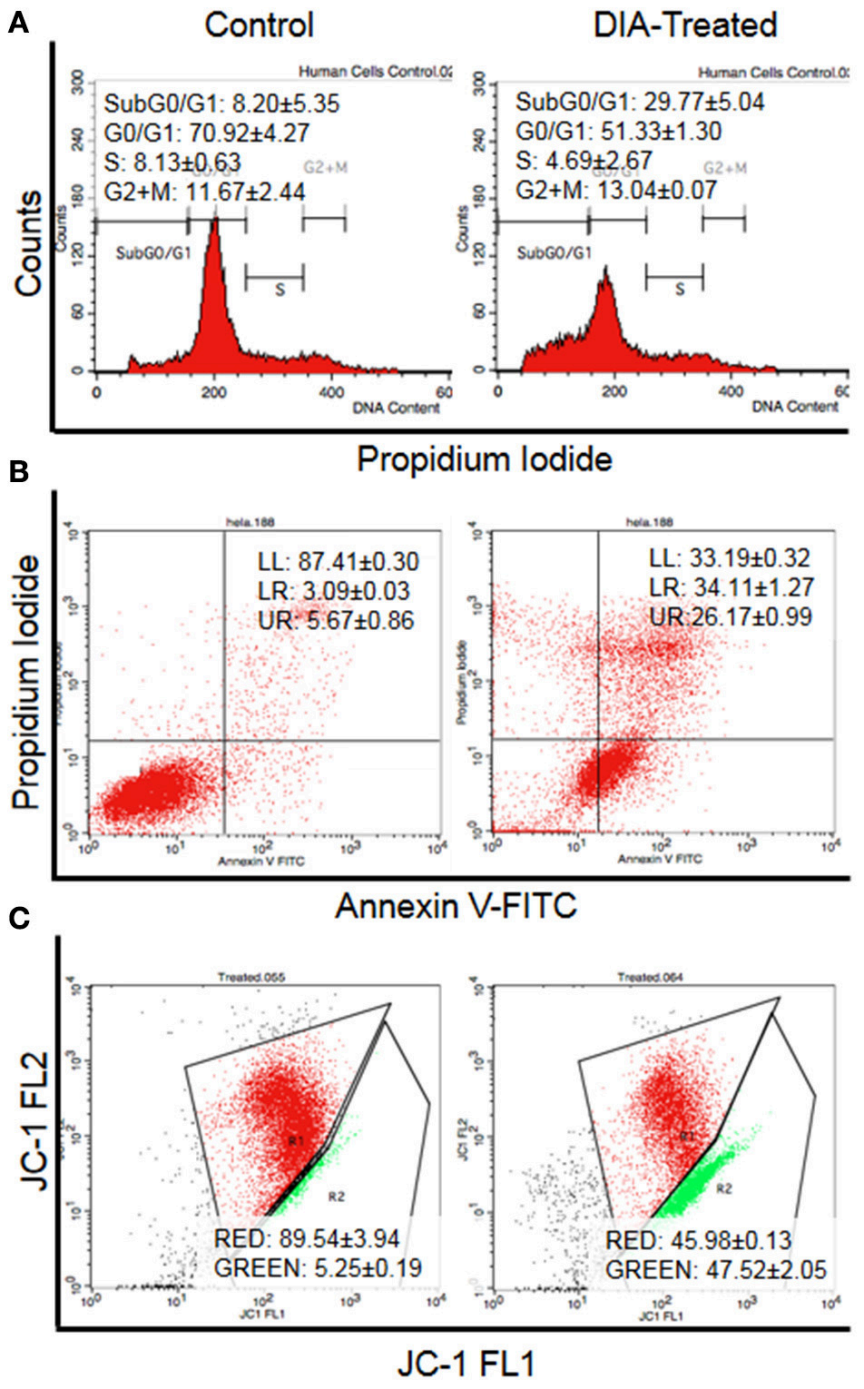
**Flow cytometry analysis of (A) Cell cycle analysis by staining the DNA using propidium iodide, (B) Annexin V analysis for the detection of externalization of phosphatidylserine (LL, viable; LR, Early apoptosis; UR, Late apoptosis), and (C) JC-1 analysis for the detecting the change of mitochondrial membrane potential in HeLa cells (Red: Aggregates; Green: Monomers) after 48 h of treatment with 15 μg/mL of DIA**.

Based on the MTT results, DIA only showed HeLa cells viability (Figure 1) in a dose-dependent manner but not on breast cancer (MCF-7 and MDA-MB231), colon cancer (HT-29), and hepatoblastoma (HepG2) cell lines (results not shown) at concentration up to 30 μg/mL. As in Figure 2, the half-maximal inhibitory concentration (IC50) of DIA against HeLa cells after 48 h was 15 μg/mL. Moreover, as in Figure 3A, the effects of DIA on HeLa cells was apparent as it induces an increase in the Sub G0/G1 phase. The percentage of cell population in the Sub G0/G1 phase for the untreated HeLa cells was 8.2%, while for the DIA-treated cells was 29.77%. Additionally, as evidenced by the Annexin V assay, DIA-treated HeLa cells had an increase in the early apoptosis and late apoptosis populations, coupled with a decrease in the viable cell population, comparing to the untreated cells. As shown in Figure 3B, the percentage of viable cells for the untreated cells was 97.97%, this was followed by a decrease to 33.19% after 48 h of treatment with DIA. For the early apoptosis population, the percentage in the untreated cells was 0.05%, while in the DIA-treated cells, the percentage of the population increased to 34.11%. A similar pattern can also be observed in the late apoptosis population, from 0.02% in the untreated cells to 26.17% in the DIA-treated cells. Furthermore, based on the JC-1 assay, the percentage of monomers in the DIA-treated cells (47.52%) was higher than the untreated cells (5.25%) as displayed in Figure 3C.

### DIA regulated cellular stress-related proteins in HeLa cells

**Figure 4:**
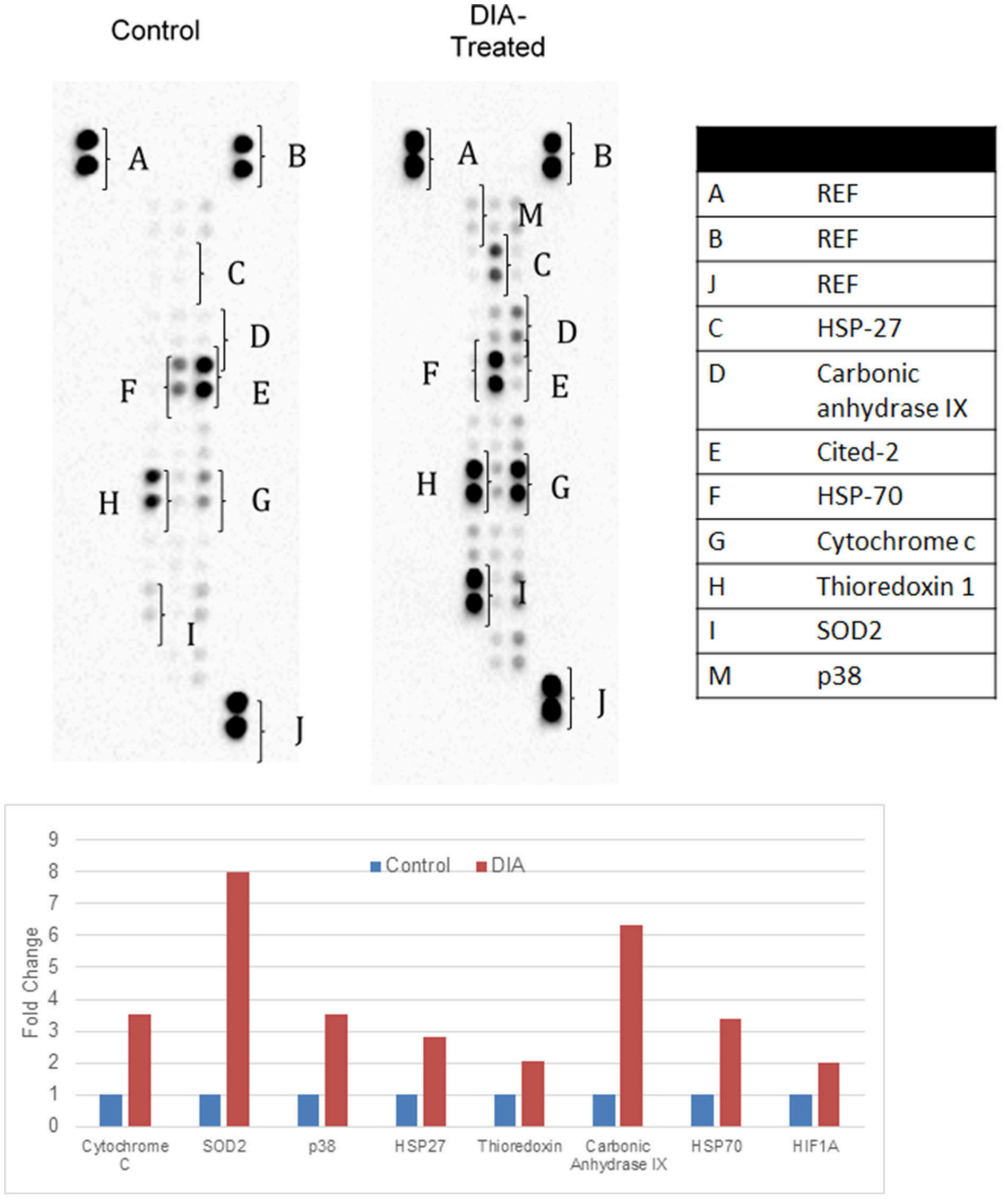
**Representative image of the proteome profiler analysis for cell stress-related images as well as the quantification values for HeLa cells; both control (untreated) and DIA-treated cells (15 ug/mL) for 48 h**.

**Figure 5:**
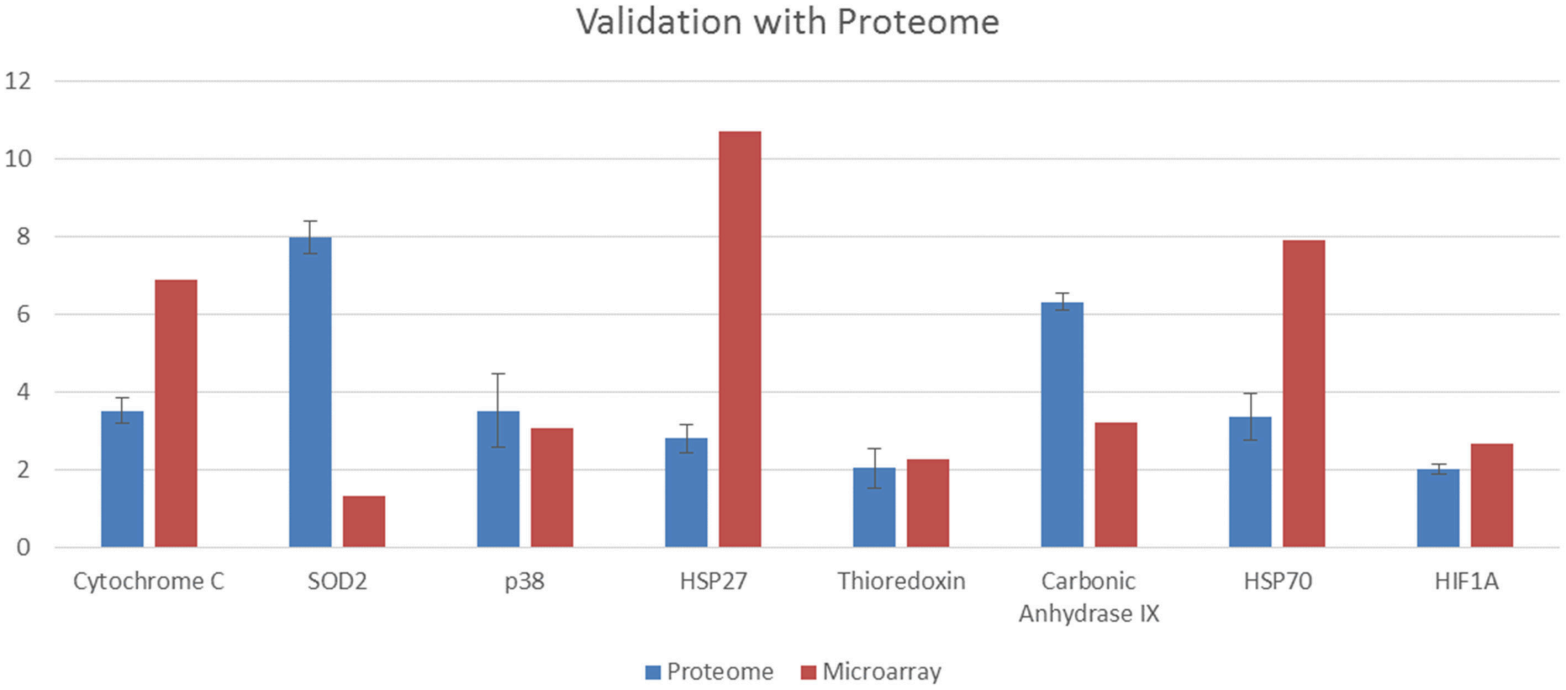
**Validation of the proteome profiler results by comparing the expression of proteins to the respective genes in microarray (***p***-value for the microarray results are ***p*** < 0.05)**.

Figure 4 illustrates the proteome profiler analysis for cell-stress related proteins as well as the quantification values. DIA-treated cells managed to increase the regulation of several heat shock proteins including hsp27 and hsp70. Moreover, the expression of cytochrome C, SOD2, thioredoxin, carbonic anhydrase IX, p38, and HIF-1a were also increased upon induction with DIA in comparison with the control. The validation of both the microarray and proteome results is presented in Figure 5. Similar pattern of expression could be seen for all of the proteins in the proteome to the same genes in the microarray differential analysis.

### DIA regulated the expression of apoptosis, oxidative stress, and chaperone-related genes

cDNA microarray study was done to determine the effects of DIA on the mRNA expression of HeLa cells. Based on Table 2, DIA managed to regulate a large number of genes related to apoptosis, oxidative stress, and heat shock proteins. DIA affected the expression of 96 genes that are involved in either cell death or cell survival.

**Table 2:** **Differentially regulated genes related to oxidative stress and MAPK pathway in HeLa cells after 48 h of treatment with 15 μg/mL DIA with ***p*** < 0.05**.

**Accession number**	**Gene symbol**	**Gene name**	**Log fold change**	**Regulation**
NM_002501	NFIX	Nuclear factor I/X (CCAAT-binding transcription factor)	2.64	Survival
NM_000499	CYP1A1	Cytochrome P450, family 1, subfamily A, polypeptide 1	2.38	Survival
NM_145791	MGST1	Microsomal glutathione S-transferase 1	7.61	Survival
NM_003998	NFKB1	Nuclear factor of kappa light polypeptide gene enhancer in B-cells 1	4.23	Survival
NM_201397	GPX1	Glutathione peroxidase 1	3.51	Survival
NM_000854	GSTT2	Glutathione S-transferase theta 2	5.67	Survival
NM_001752	CAT	Catalase	3.01	Survival
NM_002133	HMOX1	Hemeoxygenase (decycling) 1	2.44	Survival
NM_001261445	TXNRD1	Thioredoxinreductase 1	4.88	Survival
NM_001270458	MAOA	Monoamine oxidase A	1.56	Survival
NM_000101	CYBA	Cytochrome b-245, alpha polypeptide	6.63	Survival
NM_002084	GPX3	Glutathione peroxidase 3 (plasma)	–2.59^*^	Survival
NM_006440	TXNRD2	Thioredoxin reductase 2	2.04	Survival
NM_138980	MAPK10	Mitogen-activated protein kinase 10	–3.62^*^	Survival
NM_000379	XDH	Xanthine dehydrogenase	–1.48^*^	Survival
NM_016931	NOX4	NADPH oxidase 4	1.60	Survival
NM_000454	SOD1	Superoxide dismutase 1, soluble	9.92	Survival
NM_201397	GPX1	Glutathione peroxidase 1	10.58	Survival
NM_012473	TXN2	Thioredoxin 2	2.37	Survival
NM_002229	JUNB	Jun B proto-oncogene	1.05	Survival
NM_000637	GSR	Glutathionereductase	1.99	Survival
NM_005952	MT1X	Metallothionein 1X	5.07	Survival
NM_001024465	SOD2	Superoxide dismutase 2, mitochondrial	2.63	Survival
NM_006164	NFE2L2	Nuclear factor, erythroid 2-like 2	–3.82^*^	Survival
NM_001072	UGT1A6	UDP glucuronosyltransferase 1 family, polypeptide A6	2.39	Survival
NM_001025366	VEGFA	Vascular endothelial growth factor A	–5.57^*^	Survival
NM_001216	CA9	Carbonic anhydrase IX	1.67	Survival
NM_004380	CREBBP	CREB binding protein	3.67	Survival
NM_181054	HIF1A	Hypoxia inducible factor 1, alpha subunit	2.67	Survival
NM_005345	HSPA1A	Heat shock 70 kDa protein 1A	7.92	Survival
NM_001540	HSPB1	Heat shock 27 kDa protein 1	10.71	Survival
NM_000043	FAS	Fas cell surface death receptor	1.7873325	Death
NM_001226	CASP6	Caspase 6, apoptosis-related cysteine peptidase	2.8654807	Death
NM_003810	TNFSF10	Tumor necrosis factor (ligand) superfamily, member 10	–1.0368137^*^	
NM_021975	RELA	v-rel avian reticuloendotheliosis viral oncogene homolog A	1.0848213	
NM_213566	DFFA	DNA fragmentation factor, 45kDa, alpha polypeptide	1.9827862	Death
NM_138578	BCL2L1	BCL2-like 1	1.5335723	Death
NM_033306	CASP4	Caspase 4, apoptosis-related cysteine peptidase	1.2512972	Death
NM_001012271	BIRC5	Baculoviral IAP repeat containing 5	7.95958	
NM_019887	DIABLO	Diablo, IAP-binding mitochondrial protein	5.7222176	
NM_004346	CASP3	Caspase 3, apoptosis-related cysteine peptidase	5.705864	Death
NM_002467	MYC	v-myc avian myelocytomatosis viral oncogene homolog	3.997664	
NM_138764	BAX	BCL2-associated X protein	1.3814521	Death
NM_138621	BCL2L11	BCL2-like 11 (apoptosis facilitator)	1.6899183	
NM_020529	NFKBIA	Nuclear factor of kappa light polypeptide gene enhancer in B-cells inhibitor, alpha	5.042662	
NM_058197	CDKN2A	Cyclin-dependent kinase inhibitor 2A	7.5630064	
NM_032977	CASP10	Caspase 10, apoptosis-related cysteine peptidase	3.1102889	Death
NM_000639	FASLG	Fas ligand (TNF superfamily, member 6)	–2.4349697^*^	Death
NM_033355	CASP8	Caspase 8, apoptosis-related cysteine peptidase	2.0166228	Death
NM_001127184	CFLAR	CASP8 and FADD-like apoptosis regulator	4.176869	Death
NM_002392	MDM2	MDM2 proto-oncogene, E3 ubiquitin protein ligase	2.3782425	Survival
NM_000546	TP53	Tumor protein p53	1.6474063	Death
NM_021138	TRAF2	TNF receptor-associated factor 2	7.908759	Death
NM_018947	CYCS	Cytochrome c, somatic	6.6717825	Death
AK094730	HRK	Harakiri, BCL2 interacting protein	1.1366951	
NM_207002	BCL2L11	BCL2-like 11 (apoptosis facilitator)	1.0382731	Death
NM_004346	CASP3	Caspase 3, apoptosis-related cysteine peptidase	1.0135665	Death
NM_003639	IKBKG	Inhibitor of kappa light polypeptide gene enhancer in B-cells, kinase gamma	5.08775	
NM_003824	FADD	Fas (TNFRSF6)-associated via death domain	7.09289	Death
NM_181523	PIK3R1	Phosphoinositide-3-kinase, regulatory subunit 1 (alpha)	1.7552358	
NM_000043	FAS	Fas cell surface death receptor	1.2629685	Death
NM_004322	BAD	BCL2-associated agonist of cell death	6.317196	Death
NM_021138	TRAF2	TNF receptor-associated factor 2	2.5242937	Death
NM_014452	TNFRSF21	Tumor necrosis factor receptor superfamily, member 21	1.2962595	
NM_003804	RIPK1	Receptor (TNFRSF)-interacting serine-threonine kinase 1	1.8435402	
NM_000612	IGF2	Insulin-like growth factor 2	–1.0878063^*^	Survival
NM_001188	BAK1	BCL2-antagonist/killer 1	3.254304	
NM_000875	IGF1R	insulin-like growth factor 1 receptor	2.7272189	
NM_145725	TRAF3	TNF receptor-associated factor 3	–5.650675^*^	Death
NM_000657	BCL2	B-cell CLL/lymphoma 2	–2.1980934^*^	Death
AK309150	BAD	BCL2-associated agonist of cell death	2.1382089	Death
NM_197966	BID	BH3 interacting domain death agonist	3.6607141	Death
NM_002228	JUN	Jun proto-oncogene	1.9206382	Survival
NM_001289072	HELLS	Helicase, lymphoid-specific	2.2962887	
NM_033292	CASP1	Caspase 1, apoptosis-related cysteine peptidase	3.3557472	Death
NM_003842	TNFRSF10B	Tumor necrosis factor receptor superfamily, member 10b	4.5481834	Death
NM_207002	BCL2L11	BCL2-like 11 (apoptosis facilitator)	–1.1714572^*^	Death
NM_003789	TRADD	TNFRSF1A-associated via death domain	5.638668	
NM_004131	GZMB	Granzyme B (granzyme 2, cytotoxic T-lymphocyte-associated serine esterase 1)	–4.478533^*^	
NM_001229	CASP9	Caspase 9, apoptosis-related cysteine peptidase	4.309328	Death
NM_003805	CRADD	CASP2 and RIPK1 domain containing adaptor with death domain	4.569709	Death
NM_002392	MDM2	MDM2 proto-oncogene, E3 ubiquitin protein ligase	–1.910822^*^	Death
NM_004031	IRF7	Interferon regulatory factor 7	4.7208776	
NM_033338	CASP7	Caspase 7, apoptosis-related cysteine peptidase	4.725479	Death
NM_001556	IKBKB	Inhibitor of kappa light polypeptide gene enhancer in B-cells, kinase beta	3.2848263	
NM_005658	TRAF1	TNF receptor-associated factor 1	–1.2607836^*^	Death
NM_001278	CHUK	Conserved helix-loop-helix ubiquitous kinase	4.082881	
NM_003810	TNFSF10	Tumor necrosis factor (ligand) superfamily, member 10	–1.1297648^*^	
NM_181861	APAF1	Apoptotic peptidase activating factor 1	1.063245	Death
NM_001282669	DFFB	DNA fragmentation factor, 40kDa, beta polypeptide (caspase-activated DNase)	1.0859745	Death
NM_002198	IRF1	Interferon regulatory factor 1	2.7887836	
NM_002503	NFKBIB	Nuclear factor of kappa light polypeptide gene enhancer in B-cells inhibitor, beta	2.073728	
NM_197966	BID	BH3 interacting domain death agonist	6.265839	
NM_004324	BAX	BCL2-associated X protein	1.0556245	
NM_032515	BOK	BCL2-related ovarian killer	4.000063	
NM_001202519	CFLAR	CASP8 and FADD-like apoptosis regulator	2.3586068	Death

* *Negative values represent down-regulated genes upon treatment*.

### Validation of microarray results with quantitative real-time PCR

**Figure 6:**
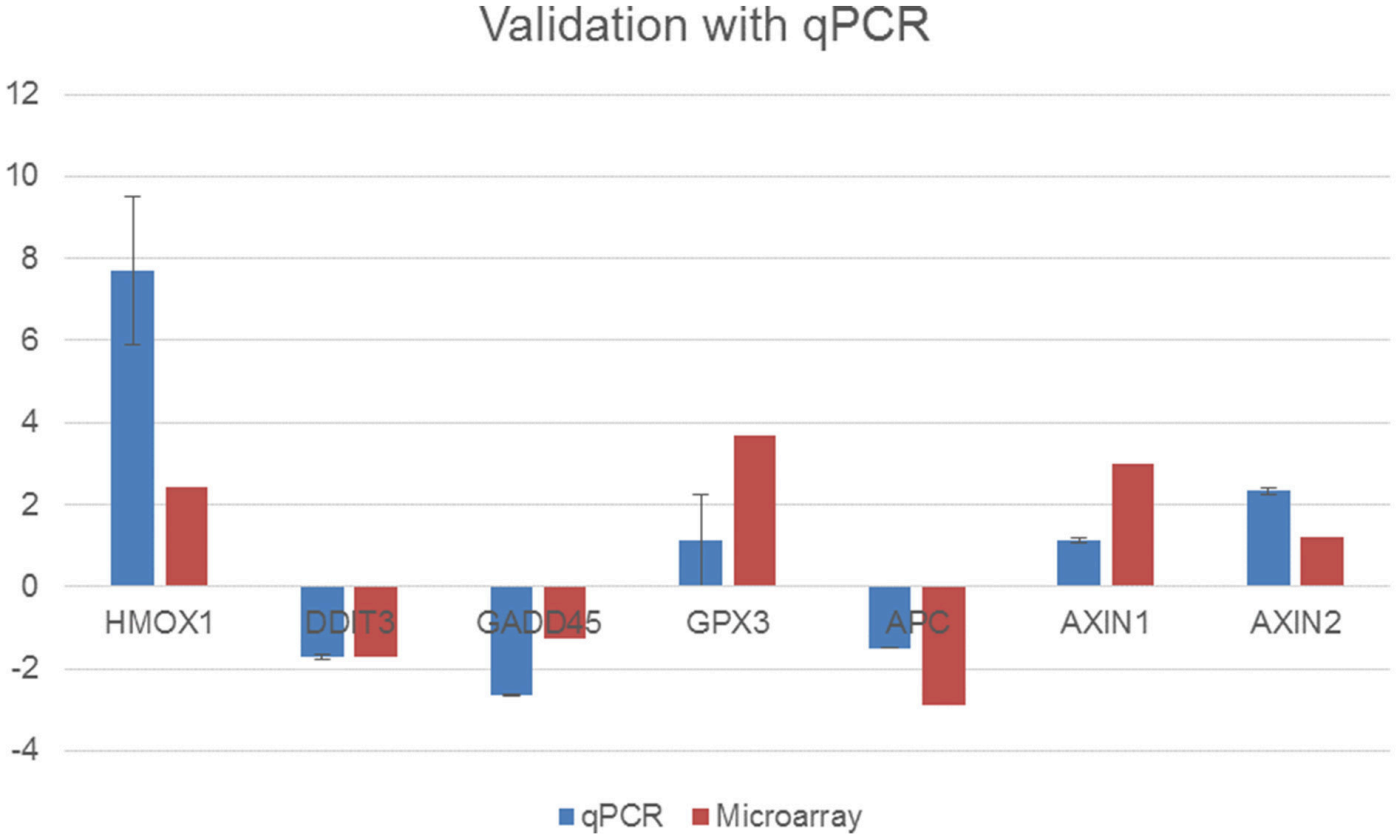
**Validation of the microarray results using quantitative real-time PCR with selected genes in total RNA samples extracted from the control cells and 15 μg/mL DIA-treated cells (***p***-value for the microarray results are ***p*** < 0.05)**.

To validate the results of differentially regulated genes from the microarray analysis, a set of genes were selected and analyzed using qPCR. All of the validated genes in qPCR had a similar pattern of expression in the microarray results, as presented in Figure 6.

### DIA-treated cells have a higher amount of SOD and GSH

**Figure 7:**
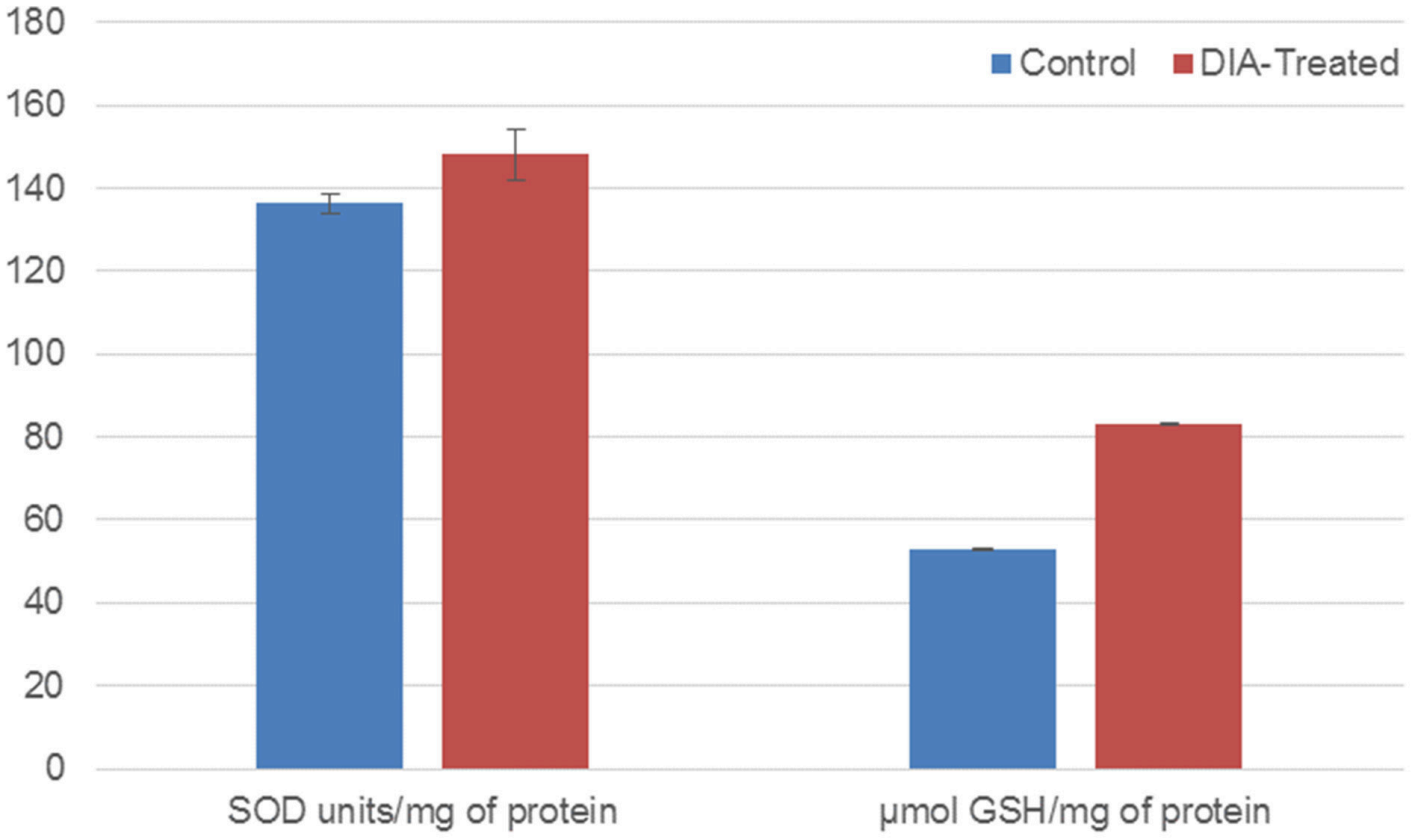
**Bar chart analysis of the SOD units/mg of protein and μmol GSH/mg of protein in control cells and DIA-treated HeLa cells after 48 h of treatment with 15 μg/mL of DIA**.

Based on Figure 7, the production of both SOD and GSH were elevated in DIA-treated cells comparing to the untreated cells (control). DIA-treated cells have a 1 fold and 1.57 fold change difference respectively from the control cells.

## Discussion

Cellular stress plays an important role in response to chemotherapeutic agents, and this has been one of the major concerns in finding the perfect treatment for cancer, even for cervical cancer (Portt et al., 2011; Kim et al., 2014). *F. Imperialis* has been long known to possess medicinal properties. The extracts of this plant have not been extensively studied on yet especially on the diterpene group. There are several notable diterpenes that possess promising anti-cancer activities such as carnosol, crispene e, and taxol (Stahlhut et al., 1999; Chun et al., 2014; Mantaj et al., 2015). To the best of our knowledge, the anti-cancer effects of DIA extracted from the bulbs of *F. Imperialis* on HeLa cells has not been reported yet.

There are various phytochemicals that possess anti-cancer properties by modulating the cellular stress pathway (Kim et al., 2014). Additionally, there are also several agents used for cancer therapy that cause cellular ROS stress including cisplatin, ascorbic acid, and emodin (Pelicano et al., 2004). The cellular stress mechanism is diverged into multiple responses and one of the responses that could be initiated is cell death, including apoptosis (Martindale and Holbrook, 2002; Fulda et al., 2010; Portt et al., 2011). Cell death can be measured through various parameters such as DNA damage through cell cycle analysis, cellular membrane changes, and mitochondrial potential changes (Schmitt et al., 2007; Branzei and Foiani, 2008). At the molecular level, based on Table 2, the activation of pro-apoptotic genes is prevalent. The expression of the FADD and FAS gene in DIA-treated cells increased comparing to the control cells. The activation of FADD and FAS triggered a downstream of execution of apoptosis-related proteins such as caspase 8, caspase 3, BID, and JNK (Gupta et al., 2004; Clarke and Tyler, 2009), which subsequently regulate the BCL2-family proteins. The BCL-2 family is crucial in a cellular response mechanism as it can contribute to the switch of cell death vs. cell survival (Gross et al., 1999; Cory et al., 2003; Schmitt et al., 2007). Pro-apoptotic BCL-2 family genes such as BAX, BAD, and BAK were increased in DIA-treated cells. These proteins can cause mitochondrial dysfunction, which in turns affect the permeability transition pore, increased in radical oxygen species (ROS) and the release of cytochrome C (Gross et al., 1999; Cory et al., 2003). Cytochrome C is a pro-apoptotic protein that recruits the activation of apaf-1 and caspase 9 (Gross et al., 1999; Cory et al., 2003). As evidenced by the increase in gene regulation of cytochrome c, apaf-1, and caspase 9, as well as the changes in the mitochondrial membrane potential in the JC-1 assay, DIA treatment induced apoptosis through the mitochondrial pathway. Based on the cell cycle analysis, DIA increased the percentage of population in SubG0/GI which suggests that the treatment may induce DNA fragmentation in the execution of apoptosis. The regulation of the AIF gene and caspase 3 may be involved in the DNA fragmentation process. All these results suggest that DIA induced apoptosis in HeLa cells through DNA fragmentation and mitochondrial membrane potential changes.

Besides cell death, cellular stress response may also trigger the cell survival motion within the cancer cells (Martindale and Holbrook, 2002; Fulda et al., 2010). Both the protein and gene expression of HSP27 and HSP70 were elevated in HeLa cells upon treatment with DIA, which suggests that the cells were attempting at recovering from the damage induced by regulating these chaperones. The heat shock protein response is a classic retaliation to stress (Feder and Hofmann, 1999; Fulda et al., 2010; Seigneuric et al., 2011; Calderwood et al., 2012). Heat shock proteins are a highly conserved set of protein chaperones that promote cell survival in stressful conditions (Calderwood et al., 2006; Seigneuric et al., 2011). These proteins are known to be overexpressed in cancer especially HSP27, HSP70, and HSP90 (Calderwood et al., 2006, 2012; Seigneuric et al., 2011). Hence, there has been development of cancer biomarkers and vaccine for these proteins for the treatment of cancer (Calderwood et al., 2006, 2012; Seigneuric et al., 2011). HSP27 and HSP70 were thought to interact in the anti-cell death process as it can inhibit cytochrome c, caspase 9 and eventually the whole apoptotic cascade (Calderwood et al., 2006; Fulda et al., 2010).

Another reaction to cell stress is the response to oxidative stress (Martindale and Holbrook, 2002; Fulda et al., 2010; Reuter et al., 2010; Portt et al., 2011). Based on the microarray and proteome results, DIA managed to induce oxidative stress in HeLa cells upon treatment. The introduction of anti-cancer agents can trigger the production of ROS substantially (Reuter et al., 2010; Fiaschi and Chiarugi, 2012). The production of ROS is a normal metabolic process in a given cellular system, nevertheless, the balance between ROS and anti-oxidants play a pivotal role in the progression of cancer (Reuter et al., 2010; Fiaschi and Chiarugi, 2012; Ma, 2013). Moreover, the hypoxic conditions of the microenvironment could also contribute to the sustain release of ROS. One of the hallmark of cancer is that cancer cells thrive under hypoxic conditions (Bartrons and Caro, 2007). This will usually lead to the activation of HIF1 protein which in turns will affect the accumulation of ROS (Bartrons and Caro, 2007; Reuter et al., 2010). Additionally, the activation of HIF1 could also affect the activation of carbonic anhydrase IX and VEGFA, which both proteins are inclined to participate in the progression of tumorigenesis (Hui et al., 2002; Jubb et al., 2004; Dungwa et al., 2011). The presence of ROS could affect both the cell death and cell survival mode. One of the pathways that is activated in an oxidative stress state is the KEAP1/NRF-2 stress pathway (Reuter et al., 2010; Gorrini et al., 2013; Ma, 2013). Moreover, the sustained production of ROS will also activate several other signaling pathways such as JNK, MAPK, and ERK pathways. The MAPK pathway could also contribute to the activation of the NRF2 pathway adding to the ROS-mediated initiation of NRF2.

**Figure 8:**
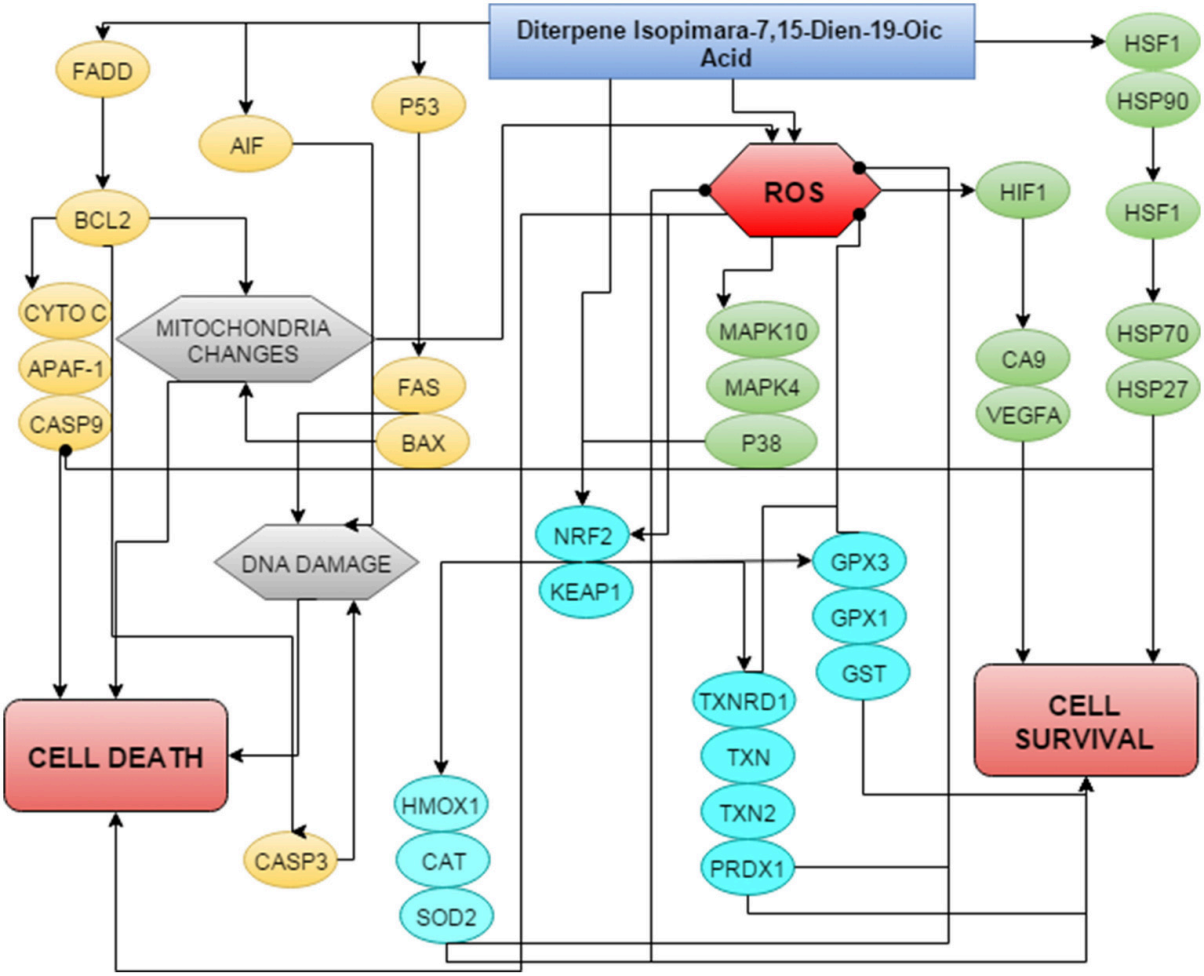
**Proposed schematic of the mechanism of action of DIA in HeLa cells by regulating both the anti- and pro-survival pathways**.

The expression of NRF2-related genes in DIA-induced HeLa cells is significant and this may give way to the underlying mechanism of DIA. The NRF2 is the key player to several antioxidant pathways including the iron sequestration pathway, quinone detoxification, GSH production, and thioredoxin production (Nguyen et al., 2009; Gorrini et al., 2013; Ma, 2013). Once the NRF2 is stimulated it will further activate phase II detoxification enzymes (Kwak and Kensler, 2010). The thioredoxin (trx) and glutathione pathway are among the antioxidants pathway that can cross-talk with multiple other pathways and with each other (Brigelius-Flohé et al., 2012; Isaac Harris et al., 2014; Lu and Holmgren, 2014; Vriend and Reiter, 2015). Both systems are dependent on NADPH and are involved in the antioxidant defensive mechanism, redox regulation and cell growth (Arnér and Holmgren, 2006; Peng et al., 2012, 2014). There are various ways as to how the thioredoxin system contributes toward the progression of cancer (Arnér and Holmgren, 2006). There are two trx systems in the mammalian cells; the cytosolic trx and mitochondrial trx (Lu and Holmgren, 2014). Both the cytosolic and mitochondrial trx systems are dependent on peroxidases and eventually involved in the redox regulation (Lu and Holmgren, 2014). Thioredoxin reductase 1 is known to be overexpressed in most malignant cancer cells (Miyazaki et al., 1998; Yoo et al., 2006; Karlenius and Tonissen, 2010). In fact, besides being involved in the defensive mechanism of cells, thioredoxin peroxidase (TRx) has been reported to inhibit apoptosis by interfering with p53 and p21 (Zhang et al., 1997; Ueno et al., 1999; Brigelius-Flohé et al., 2012). Targeting players of the thioredoxin pathway such as thioredoxin reductase, peroxidase or thioredoxin has been an interest for cancer therapy (Arnér and Holmgren, 2006; Lu et al., 2007; Karlenius and Tonissen, 2010; Penney and Roy, 2013). Moreover, in most cancer cells, the level of GSH is often upregulated and can contribute to the drug-resistance mechanism (Balendiran et al., 2004; Traverso et al., 2013). GSH is a non-protein molecule that has several important physiological properties (Balendiran et al., 2004). Moreover, it is known that GSH can contribute toward the initiation of cancer. In phase II detoxification process, GST plays an important role as it assists in the conjugation of GSH with different cancer-promoting electrophiles (Balendiran et al., 2004). The high levels of GSH and GST has become one of the important properties in various types of cancer (Balendiran et al., 2004). Besides GSH, the level of SOD was also increased in DIA-treated cells. SOD is known to be higher in cancer cells than normal cells (Oberley and Buettner, 1979). The role of SOD as an antioxidant is by converting the radical O_2_ to the less radical H_2_O_2_ (Matés, 2000; Kowald and Klipp, 2004; Valko et al., 2006). Catalase, another antioxidant enzyme will later on convert the produced H_2_O_2_ to water and oxygen (Matés, 2000; Kowald and Klipp, 2004; Valko et al., 2006;). Additionally, the GPx enzyme will also convert H_2_O_2_ to water and GSSG (Matés, 2000; Valko et al., 2006). Another important phase II detoxification enzyme is the pro-survival HMOX-1, which is heavily involved in the inactivation of the pro-oxidant heme to ferrous iron, carbon monoxide and bilirubin (Lau et al., 2008; Yim et al., 2011). Activation of heat shock protein and antioxidant mechanism by this diterpene may protect the cancer cell and thus reduce the killing efficacy of DIA. Overall, Figure 8 summarizes the proposed schematic of mechanism of action of DIA in HeLa cells.

## Conclusion

Overall, DIA managed to induce cellular stress in HeLa cells as evidenced by the results above. DIA induced cellular death via the apoptosis pathway by regulating the FAS and BCL-2 family genes. On the other hand, DIA also activated several pro-survival pathways including the heat shock protein response and anti-oxidant pathways. The dual regulation of DIA in HeLa cells could further benefit the understanding to the molecular mechanism of DIA. Future work using this compound can be applied in an *in vivo* setting to promote a deeper understanding of the function of DIA as an anti-cancer agent.

## Author contributions

NA, SY, NBA: Designed the experiments. NA, AM, MN, NZ, SY: Performed the experiments. MN, SZ, JI: Preparation and identification of the compound. SY, NA, NR: Analyzed the results. NA, SY, MN: Prepared the manuscript.

### Conflict of interest statement

The authors declare that the research was conducted in the absence of any commercial or financial relationships that could be construed as a potential conflict of interest.
